# Case report: hydroxychloroquine infiltrative cardiomyopathy

**DOI:** 10.1093/ehjcr/ytaf052

**Published:** 2025-01-30

**Authors:** Miguel Ángel Silva Cerpa, María Victoria Mogollón Jiménez, Paloma Gil Bernabé, Juan Carlos Rama Merchán

**Affiliations:** Cardiology Department, Hospital Universitario de Cáceres, Av. de la Universidad, 75, 10004 Cáceres, Spain; Cardiology Department, Hospital Universitario de Cáceres, Av. de la Universidad, 75, 10004 Cáceres, Spain; Pathological Anatomy Department, Hospital Puerta del Hierro de Majadahonda, C. Joaquín Rodrigo, 1, 28222 Majadahonda, Madrid, Spain; Cardiology Department, Hospital Universitario de Cáceres, Av. de la Universidad, 75, 10004 Cáceres, Spain

## Case description

**Figure 1:**
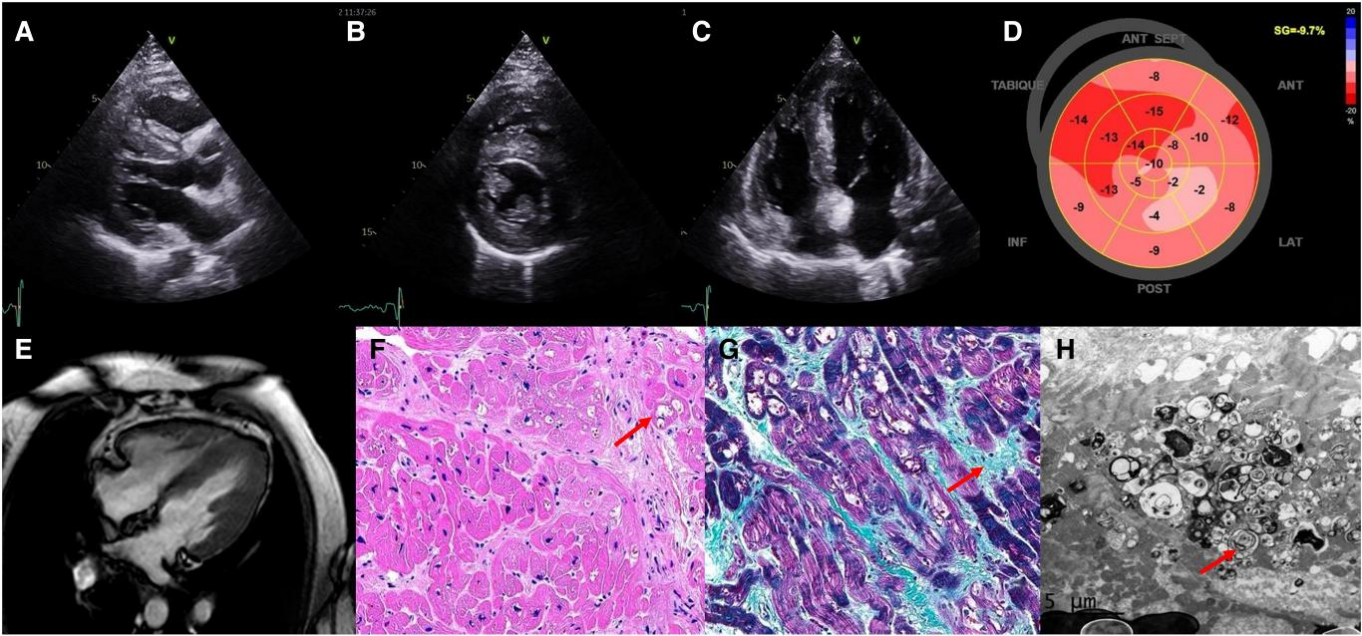
The figure shows 2D echocardiographic (*A–C*) and cardiac magnetic resonance (*E*) study showing severe biventricular hypertrophy and decreased global longitudinal strain (*D*). Furthermore, in the pathological anatomy images of the endomyocardial biopsy: presence of vacuoles in the cytoplasm of some fibres (*F*, arrow), interstitial fibrosis in Masson’s trichrome (*G*, arrow), and electron-dense, lamellar, and concentric inclusions of pseudomyelinoid morphology in the cytoplasm by electron microscopy (*H*, arrow). All of this is compatible with chloroquine–hydroxychloroquine toxicity, pathognomonic after ruling out other causes of lysosomal storage disease.^1,2^

A 46-year-old female with history of systemic lupus erythematosus, with pulmonary vascular hypertension (Group 1), treated with hydroxychloroquine (HCQ), prednisone, and mycophenolate mofetil, presented at the emergency department with worsening of her dyspnoea (minimal effort) and limb oedema. The transthoracic echocardiography showed a severe biventricular hypertrophy with an interventricular septum of 21 mm (*Figure 1A* and *B*) and interatrial septum hypertrophy with a normal left ventricular function (*Figure 1C*) and decreased global longitudinal strain (*Figure 1D*) (previous of −16.1%). All of this was suggestive of infiltrative cardiomyopathy; therefore, the study was completed with a cardiac magnetic resonance, which confirmed the biventricular hypertrophy with large areas of intramyocardial gadolinium enhancement in late sequences in both ventricles (*Figure 1E*). The bone scintigraphy with Tc99 and measurement of light chains in blood/urine were negatives. The genetic study with a complete panel for hypertrophic cardiomyopathy was negative. Finally, an endomyocardial biopsy was performed, observing granular inclusions with vacuolation of cardiomyocytes (*Figure 1F*), intravacuolar lamellar bodies and curvilinear bodies with interstitial fibrosis with Masson’s trichrome technique (*Figure 1G*) and with the electron microscope (*Figure 1H*), confirming the diagnosis of infiltrative cardiomyopathy due to HCQ. The withdrawal of HCQ was associated with clinical, analytical, and echocardiographic improvement, especially the level of cardiac markers (natriuretic peptides) in the laboratory tests at the 1-year follow-up.

## Data Availability

The data underlying this article are available in the article and in its online supplementary material.
